# Butterflies visit more frequently, but bees are better pollinators: the importance of mouthpart dimensions in effective pollen removal and deposition

**DOI:** 10.1093/aobpla/plw001

**Published:** 2016-01-07

**Authors:** Beyte Barrios, Sean R. Pena, Andrea Salas, Suzanne Koptur

**Affiliations:** 1 Department of Biology, Florida International University, Miami, FL 33199, USA; 2 Present address: Rare Plant Conservation Program, Bok Tower Gardens, Lake Wales, FL 33853, USA; 3 Present address: Ecology and Evolutionary Biology, University of California, Irvine, Irvine, CA, USA

**Keywords:** Apocynaceae, floral visitors, Hymenoptera, Lepidoptera, pine rocklands, pollen transfer efficiency, pollination

## Abstract

Pollination studies often use visitation frequency of potential pollinators as an indicator of their importance, but this is only one component and may not reflect actual pollen transfer rates. In this study, we determine the most effective pollinator group of *Angadenia berteroi*, a tropical perennial subshrub with large yellow flowers that set few fruits. We determined visitation frequency and pollen transfer effectiveness of the four most common groups of visitors (long- and short-tongued bees, and skipper and non-skipper butterflies). Using potted plants, we exposed flowers to single visits from different types of pollinators to measure fruit set. We demonstrate that *A. berteroi* is most effectively pollinated by long-tongued bees, though many other species visit the flowers; the most frequent visitor group is not the most important pollinator, because they neither carry nor deposit much pollen, as the width of their proboscis is small compared with long-tongued bees. In this system, the width of the proboscis of the pollinators correlates with pollen transfer efficiency. Our results demonstrate the importance of pollen removal, pollen deposition, and fruit set, in determining the most effective pollinators, rather than visitor frequency. The distinctive morphology of these flowers, with a large bell and a narrow, short tube, suggests that other flowers of this shape may similarly benefit more from visitors with mouthparts shorter than previously considered optimal.

## Abstract (precis)

*Angadenia berteroi* is a charismatic wildflower species, native to south Florida pine rocklands, and ubiquitous in this imperiled, fire-successional habitat. We used new approaches to understand the pollination biology of the pineland golden trumpet. In this system, the width of the proboscis of the pollinators correlates with pollen transfer efficiency, and long­tongued bees are the most effective pollinators, though many other species visit the flowers. The distinctive morphology of these flowers, with a large bell and a narrow, short tube, suggests that other flowers of this shape may similarly benefit more from visitors with mouthparts shorter than previously considered optimal.

## Introduction

The evolution and diversification of the perianth has been associated with pollinator attraction (Proctor *et al*. 1996; Inouye and Kearns 1997; Richards 1997) and mechanical fit (Fenster *et al*. 2004). Pollination syndromes are groups of floral traits present in distantly related plants that share similar pollinators: floral morphology, phylogenetic position and floral reward characteristics are all important in predicting what pollinates a given plant species (Johnson and Steiner 2000; Etcheverry and Alemán 2005; Ollerton *et al*. 2007). The pollination syndrome concept reflects selection response to a functional group of pollinators that can exert similar selective pressures collectively on floral design (Fenster *et al*. 2004). Within communities, the majority of plant species are visited by a variety of pollinator groups, but visitation does not necessarily imply pollination; not all flower visitors are important and effective pollinators (Stebbins 1970; Waser *et al*. 1996; Fenster *et al*. 2004; Ne'eman *et al*. 2010). A pollinator's overall importance to a plant's reproduction involves its efficiency (successful dispersal of pollen grains deposited on conspecific stigmas) and visitation frequency (Armbruster 1985; Waser *et al*. 1996; Armbruster *et al*. 2000; Reynolds and Fenster 2008; Reynolds *et al*. 2009).

Several studies have reported that the body structure of floral visitors, especially the feeding apparatus associated with the dimensions and the morphology of the flowers, is one of the factors determining which visitors can effectively function as pollinators (Inouye 1980; Waser *et al*. 1996; Anderson *et al*. 2002; Castellanos *et al*. 2004; Ibanez 2012; Moré *et al*. 2012; Miller *et al*. 2014). Proboscis length is an important determinant of pollination efficiency during foraging for bumblebees (Inouye 1980; Dohzono *et al*. 2004; Arbulo *et al*. 2011). In hawkmoth-pollinated plants, floral tube length determines which species may transfer pollen; hawkmoths with tongues that are too short or too long will not pick up pollen effectively (Alexandersson and Johnson 2002; Anderson *et al*. 2002; Moré *et al*. 2012). Flower width has also been associated with pollen transfer and pollination efficiency (Galen 1989; Campbell *et al*. 1996).

**Figure 1.**
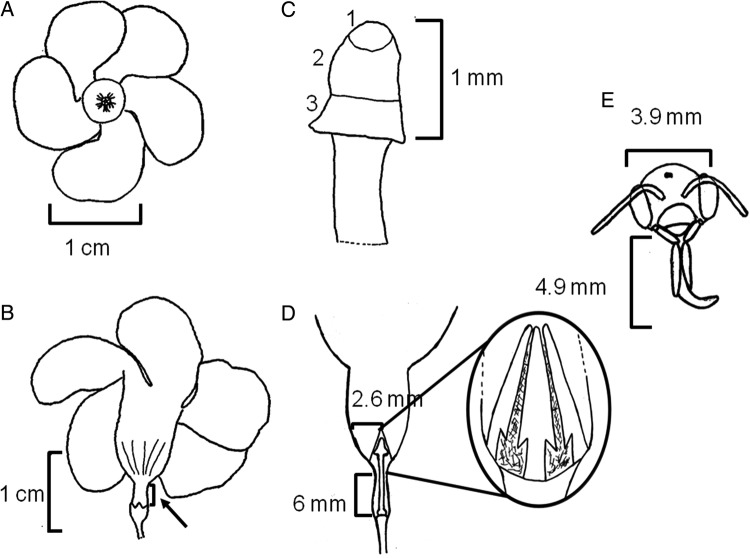
Diagram of *A. berteroi* flower. (A) Lateral view of the whole flower. (B) Abaxial view of the whole flower. Arrow points to floral tube constriction (from Barrios and Koptur 2011). (C) Style head. (1) Apical part; (2) medium secretory area and (3) receptive area. (D) Longitudinal section of the flower, and enlarged view of the anthers. (E) Diagram of the head of a bee showing how the head width and the proboscis length were measured.

**Figure 2.**
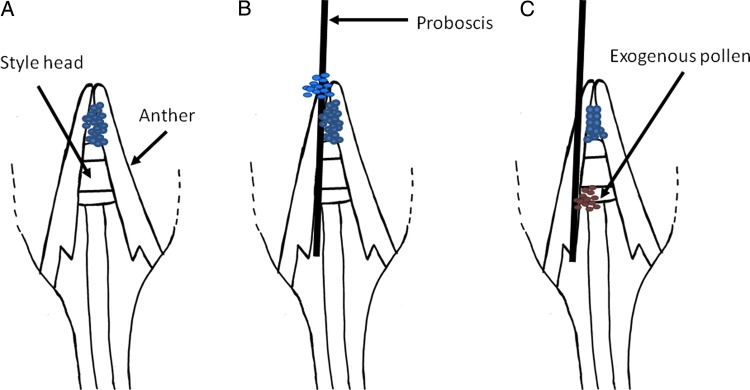
(A) Longitudinal section of flower showing pollen deposited by dehisced anther on the style head, forming the pollen chamber. Arrows point to the middle of the style head and the anthers. (B) Proboscis of the pollinator bearing exogenous pollen inserted into the floral tube. (C) As the mouthparts are retracted, exogenous pollen is deposited into the receptive area of the style head. The proboscis may also contact glue-applying midsection of style head and pick up pollen from flower just visited (not shown).

*Angadenia berteroi* (Apocynaceae, Apocynoideae) is a perennial subshrub listed as threatened by Florida's Department of Consumer Services, Division of Plant Industry (Gann *et al*. 2002). Its tubular flowers have a complex flower structure (Fig. 1); unlike asclepioid Apocynaceae that disperse large numbers of pollen grains together in pollinia, these flowers have a mechanism to glue and disperse many pollen grains onto the mouthparts of floral visitors (Fig. 2). The anthers form a conical structure surrounding the style head, depositing their pollen on the non-receptive distal portion of the style head; the receptive stigma is at the base of the style head, and the glue-bearing area is in the middle of the style head (Galetto 1997; Lipow and Wyatt 1999; Fig. 1). This type of secondary pollen presentation suggests a somewhat specialized pollination system (Yeo 1993; Torres and Galetto 1999). Flowers in the Apocynaceae have apparently evolved to attract insects with mouthparts (or other body parts) long enough to reach the nectar (Endress 1994; Proctor *et al*. 1996; Darrault and Schlindwein 2005; Pinto *et al*. 2008). Lengths of the proboscides of the pollinators are apparently related to the lengths of the floral tubes (Proctor *et al*. 1996), affecting the mechanical fit between floral tube and pollinators as well as pollination efficiency (Nilsson 1988; Boberg *et al*. 2014).

The unusual flowers of the Apocynaceae vary in floral mechanisms (Endress and Bruyns 2000), and insects are the major floral visitors of Apocynaceae s.l. (Endress 1994), with reports of beetles, butterflies, hawkmoths, flies, wasps and bees attracted to and pollinating species of this family (Haber 1984; Lopes and Machado 1999; Darrault and Schlindwein 2005; Moré *et al*. 2007; Theiss *et al*. 2007; de Moura *et al*. 2011; Wiemer *et al*. 2012; de Araújo *et al*. 2014).

Having observed a variety of visitors to *A. berteroi*, we undertook this study to determine which floral visitors are the most effective pollinators.

We address the following questions: (i) What is the diversity and abundance of animals visiting the flowers? (ii) Are all visitors equally effective at transferring pollen to conspecifics? And if not, why? We used a variety of methods to determine the effectiveness of each type of floral visitor in terms of pollen removal, simulated pollen deposition and fruit set.

## Methods

### Study species

*Angadenia berteroi* bears large, showy, yellow, tubular flowers. They have the typical complex floral arrangement of the non-asclepiad Apocynaceae (Barrios and Koptur 2011). The flowers have no notable fragrance, and offer viscous nectar as a pollinator reward, with the sugar concentration of the nectar ranging from 30 to 67 % (Barrios and Koptur 2011). Flowering begins in early April in South Florida and continues until late June; the flowers open early in the morning (prior to sunrise) and last <24 h (Barrios *et al*. 2011). Our field observations revealed that the natural level of fruit set in *A. berteroi* is low: average fruit set at six sites ranged from 3.3 to 26.4 % (16.6 % on average). The species is self-incompatible relying on pollinators to set fruit (Barrios and Koptur 2011). Though visitors were observed infrequently, Lepidoptera were by far the most common.

### Study sites

We conducted our field studies of *A. berteroi* in four pine rockland fragments and one fire management unit in Everglades National Park. We chose the sites based on the presence of many individuals of the study species (Barrios *et al*. 2011).

### Flower visitors

We conducted pollinator watches weekly, for 3 h per week per site (12 intervals of 15 min per day), from 9:00 am to 12:00 pm (the hours with the highest visitation rates, B. B. Roque, personal observations) during the flowering period (April–June). We haphazardly selected plants with open flowers for each of the intervals. Flower visitors were counted and some representatives of each group were captured at all sites using aerial nets. Floral visitors were divided into four groups, namely long-tongued bees (Apidae and Megachilidae), short-tongued bees (Halictidae), skipper (Hesperiidae) and non-skipper butterflies (Pieridae and Nymphalidae). We identified each visitor to functional pollinator group and, where possible, to species and the groups were compared (Table 1). Voucher specimens will be archived at the Florida State Collection of Arthropods (Gainesville, FL).

**Table 1.** Flower visitors of *A. berteroi* observed in the study sites, and presence/absence of pollen on the proboscis.

Visitor type	*n*	Scientific name	Pollen on proboscis
Long-tongued bee	13	*Megachile georgica*	Yes
Long-tongued bee	4	*Melissodes communis communis*	Yes
Short-tongued bee	2	*Augochlorella gratiosa*	No
Short-tongued bee	4	*Augochloropsis anonyma*	No
Non-skipper butterfly	1	*Ascia monuste phileta*	No
Non-skipper butterfly	4	*Agraulis vanillae nigrior*	Yes
Skipper	3	*Asbolis capucinus*	No
Skipper	1	*Cymaenes tripunctus*	No
Skipper	2	*Hylephyla phyleus*	No
Skipper	2	*Lerema accius*	No
Skipper	13	*Polites baracoa baracoa*	No
Skipper	5	*Wallengrenia otho*	No

Pollen grains were collected from the insect bodies to see whether visitors carried *A. berteroi* and/or other pollen. The percentage of individuals in each visitor group with pollen on the proboscis was estimated. To determine the identity of pollen grains, we used our reference collection of pollen from pine rockland plants.

To quantify the efficiency of each visitor group, we estimated pollen on the visitor mouthparts as the average number of pollen grains per individual visitor of each group. Pollination watches were performed to monitor flower visitors on plants in five different sites where *A. berteroi* is present, to provide a representation of visitors over the natural distribution of this species. Foraging behaviour was categorized by following visitor movements after they visited *A. berteroi* flowers. Visitation frequency of the floral visitors was estimated by counting the number of visits of each of the visitor groups to *A. berteroi* flowers during the observation periods where at least one visitor was seen, and calculating the corresponding percentage of the total visits observed in those periods. (Periods where no visitors were observed to flowers were not recorded, so standard frequency was not calculated.) Time spent on a flower was clocked for each insect visitor observed during our watches.

Visitation frequency and pollen on the visitor mouthparts allowed us to rank the importance of each visitor species to the reproduction of *A. berteroi* (visitation frequency × pollen on the visitor mouthparts, as modified from Reynolds and Fenster 2008). We were unable to accurately calculate pollinator importance confidence intervals as we only estimated the relative frequency data (see above).

We used the Kruskal–Wallis test to detect differences among visitor groups for the average length of the visit, as the data were not normally distributed, and then used the Mann–Whitney test (*post hoc*) to determine differences among the groups. We used the sequential Bonferroni correction to control type I error for all pairwise comparisons. Statistical analyses were performed using SPSS 21 (SPSS 2014).

### Pollinator effectiveness

We wished to determine how the thickness of the proboscides of each of the visitor groups affects pollen transfer efficiency. We first measured the length and width of the proboscis of each captured flower visitor using a dissecting microscope (Leica MZ12 5). We then used monofilament nylon fishing line of four different diameters (4 lb, 0.20 mm diameter; 6 lb, 0.23 mm diameter; 8 lb, 0.28 mm diameter and 25 lb, 0.53 mm diameter) inserted into single flowers to simulate flower visits. The different diameters were chosen to approximate the average size of the mouthparts of the four different pollinator groups (from narrowest to widest: skippers, non-skipper butterflies, short-tongued bees and long-tongued bees). Each simulated visit consisted of inserting a single 4-cm-long piece of fishing line into the corolla tube of a fresh flower until it reached the bottom of the tube (potentially contacting the middle portion of the style head where it might contact the style's sticky secretions), followed by its careful withdrawal to prevent dislodgment of any adhering pollen grains; the number of pollen grains adhering to the line was then counted. Fifty simulated visits were performed for each line diameter. This was to test for a possible relationship between the numbers of pollen grains removed and the thickness of the fishing line.

To examine a possible relationship between line thickness and pollen deposition, we hand pollinated fresh flowers using fishing line of four different diameters. Each thickness was inserted into a fresh flower to the bottom of the corolla tube to collect pollen (as above); we then stained entire length of the fishing line with methylene blue to stain the adhering pollen grains and introduced the stained portion into another new fresh flower. Flowers thus hand-pollinated were then carefully dissected, and the length of the stigmatic surface that was stained on the second (recipient) flower was recorded. This measurement (length of the stigmatic surface stained blue) was indicative of the proportion of the stigmatic surface touched by the pollen adhered to the fishing line and, by inference, the potential for pollen deposition on the stigma. We performed 23 replicates for each line diameter.

Based on our observations, both long-tongued and short-tongued bees were the only pollinator groups that consistently entered the bell of the flower to insert their proboscides into the pollen chamber. To estimate how far into the corolla tube these two types of pollinators could reach, we measured the distance from the apical part of the pollen chamber to the corolla walls using a Bausch & Lomb measuring magnifier (Fig. 1) for flowers from 30 individual plants (63 flowers total). We also measured the width of the head of the two bee groups (*n* = 4 for short-tongued bees and *n* = 17 for long-tongued bees) using a dissecting microscope.

To determine the most effective pollinator, we placed 15 greenhouse-grown potted plants in the field to quantify pollination success at Site 3, the site with the highest visitation frequency (B. B. Roque, personal observations). On 20 different days during the flowering period, we compared the qualitative effectiveness of the different pollinator groups by allowing a single visit to individual flowers on the potted plants. Flowers that were ready to open prior to observation periods were bagged, while in bud, to exclude visitors. At the time of observation (from 9:00 am to 12:00 pm), bags were removed and flowers exposed to foraging insects. For a specific flower, pollinator visits were restricted to a single visit by one individual from one of the four groups of pollinators. After visitation, a flower was labelled (by pollinator group) and bagged to exclude subsequent visitors. Pollination success was quantified for these same flowers by recording the fruit production of visited flowers on the potted plants, maintained in the greenhouse. We placed at least 15 plants per day, each with one to three open flowers, over 20 days of observations, using a new set of plants each day. We recorded a total of 69 visits to over 400 flowers observed in this potted plant placement experiment. Pollination efficiency of each visitor group was assessed by comparing the percentage of visited flowers that produced fruit after a single visit.

Analysis of variance was used to test for differences among visitor groups for proboscis length and width, and length of the stigmatic surface stained with methylene blue; *post hoc* tests were conducted using Tukey's honestly significant difference to test for differences between pairs of visitor groups. Pollen loads on the fishing line were compared using the Kruskal–Wallis test, with the Mann–Whitney test (*post hoc*) to determine differences in pollen loads among fishing line sizes, as the data were not normally distributed. We also evaluated the differences between the two groups' head widths using Student's *t*-test. We used the sequential Bonferroni correction to control type I error for all pairwise comparisons. Statistical analyses were performed using SPSS 21 (SPSS 2014).

Pollination efficiency and visitation frequency allowed us to rank the significance of each visitor group to the reproduction of *A. berteroi*. Along with fruit set from flowers visited, we determine the most effective pollinator of *A. berteroi*.

## Results

### Flower visitors

We observed a total of 153 insect visits to *A. berteroi* flowers. We captured 56 insect visitors at the 4 sites, belonging to 12 species in the orders Lepidoptera and Hymenoptera (Table 1). Within each of these groups, there were two subgroups: Lepidoptera were skippers (Hesperiidae) and non-skipper butterflies (Pieridae and Nymphalidae) and Hymenoptera were long-tongued (Megachilidae and Apidae) and short-tongued (Halictidae) bees. Skippers were the most frequent visitors, followed by long-tongued bees (Table 2). Lepidoptera were often observed to visit more than one flower on a plant, whereas long-tongued bees were much less likely than non-skipper butterflies and skippers to either visit consecutive *A. berteroi* flowers on the same plant or to return to a previously visited flower. The duration of visits differed significantly among pollinator groups (Kruskal–Wallis test, $\chi_{3}^{2}=12.3,$ *P* = 0.006, *n* = 153, Table 2).

**Table 2.** Percentage of visits and foraging behaviour of *A. berteroi* visitors. *n*, sample size. Lengths of visits with the same letters are not significantly different with Kruskal–Wallis analysis.

Visitor group	*n*	Percentage of total visits	Percentage returned to the same flower	Percentage moved to another *A. berteroi* flower	Average length of visit (s)
Long-tongued bees	45	29.4	0	15.6	7.8 ± 4.9^a^
Short-tongued bees	26	16.9	3.8	7.6	6.7 ± 3.3^a^
Non-skipper butterflies	15	9.8	26.7	33.3	9.0 ± 14.4^a^
Skippers	67	43.8	14.3	26.9	10.0 ± 5.9^b^

Long-tongued bees were the only visitors that carried large quantities of pollen on their proboscides; 76.5 % of all captured long-tongued bees carried many pollen grains on their mouthparts (Table 3). In all cases, the number of pollen grains carried by long-tongued bees (82.9 ± 69.8) was in excess of the number of ovules (61.3 ± 15 ovules per flower; B. B. Roque, personal observation). Additionally, the pollen adhered to the proboscides of the visitors in aggregated clumps that were difficult to dislodge from the insect's proboscis during manipulation. The few non-skipper butterflies we found carrying pollen had proboscides with only few pollen grains (Table 3). Long-tongued bees were the most important pollinators of *A. berteroi*, with higher estimates of pollinator importance than non-skipper butterflies.

**Table 3.** Percentage of flower visitors with pollen on the proboscis. Pollinator importance was calculated as visitation rate × pollen removal.

Visitor group	*n*	Percentage with pollen on the proboscis	Average number of pollen grains	Pollinator importance
Long-tongued bees	17	76.5	82.9 ± 69.8	2437.26
Short-tongued bees	6	0	0	0
Non-skipper butterflies	5	40.0	2.6 ± 4.3	25.48
Skippers	26	0	0	0

### Pollinator effectiveness

**Figure 3.**
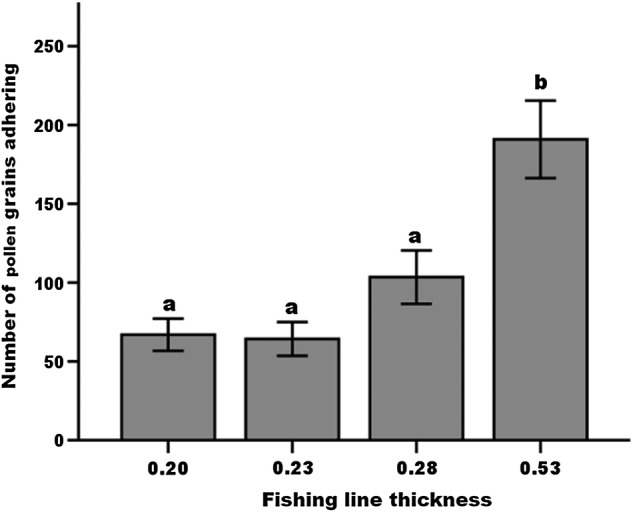
Mean and standard error of the number of pollen grains on fishing line of increasing width inserted into flowers of *A. berteroi*. The diameter of 0.20 mm approximates the diameter of the proboscis of skippers, 0.23 mm represents non-skipper butterflies, 0.28 mm represents short-tongued bees and 0.53 mm represents long-tongued bees. Sample size = 50 in each group. To aid interpretation, the number of ovules ranges from 46 to 76 ovules per flower. Diameters with the same letters are not significantly different with Kruskal–Wallis analysis.

Proboscis width and length differed significantly among visitors groups (*F*_3, 51_ = 41.11, *P* < 0.0001; *F*_3, 44_ = 85.3, *P* = 0.002, respectively, Table 4). Non-skipper butterflies and skippers had the longest proboscides (Table 4). Long-tongued bees were the visitor group with the widest proboscides (0.70 mm, Table 4); within this group, the two species of long-tongued bees (*Megachile georgica* and *Melissodes communis communis*) did not differ significantly in proboscis width or length (*t* = 1.12, df = 15, *P* = 0.26; *t* = −1.24, df = 11, *P* = 0.24, respectively). In the visitation simulations using nylon fishing line of different diameters, pollen quantity differed significantly among the widths (Kruskal–Wallis test, *P* < 0.001, *n* = 172, Fig. 3), with the widest fishing line having the greatest pollen load. There was no significant difference in the number of pollen grains extracted in simulated visits of the three other diameters, using fishing line sizes 0.20, 0.23 and 0.28 mm, which represented the proboscides of the skippers, butterflies and short-tongued bees, respectively.

**Table 4.** Proboscis measurements (mean and standard error) for flower visitors to *A. berteroi*. Proboscis width and length with the same letters are not significantly different with Tukey comparisons.

Visitor group	*n*	Proboscis width (mm)	Proboscis length (mm)
Long-tongued bees	17	0.70 ± 0.15^a^	4.95 ± 1.14^a^
Short-tongued bees	4	0.28 ± 0.29^b^	1.88 ± 0.79^a^
Non-skipper butterflies	4	0.22 ± 0.56^b^	11.39 ± 0.08^b^
Skippers	26	0.21 ± 0.16^b^	8.52 ± 4.73^b^

**Figure 4.**
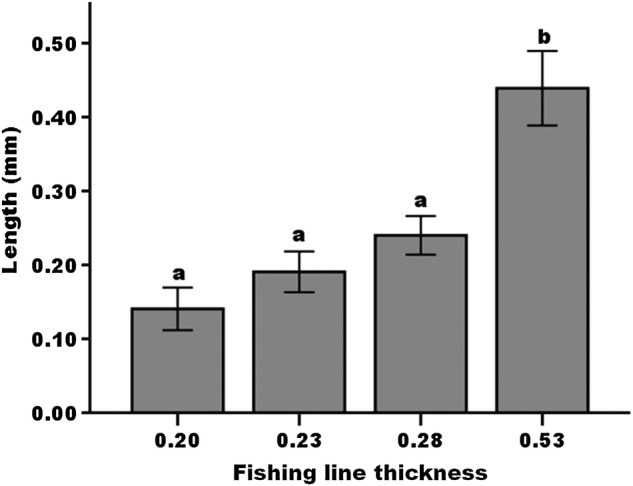
Mean and standard error of the length of the stigmatic surface stained with methylene blue. The diameter of 0.20 mm approximates the diameter of the proboscis of skippers, 0.23 mm represents non-skipper butterflies, 0.28 mm represents short-tongued bees and 0.53 mm represents long-tongued bees. Sample size = 23 per group. Diameters with the same letters are not significantly different with Tukey comparisons.

In the pollen deposition simulation experiment, the length of the stigmatic surface stained with methylene blue was also influenced by the diameter of the fishing line (*F*_3, 51_ = 14.19, *P* < 0.0001). Size 0.53 fishing line contacted a significantly larger proportion of the stigmatic surface than the smaller diameter lines (Fig. 4).

The mean (±SD) distance from the apical portion of the pollen chamber and the corolla walls was 2.6 (±0.4)mm. The mean width of the long-tongued bees' heads was 3.9 (±0.22) mm (Fig. 1), while that of short-tongued bees' heads (Halictidae) was 1.8 (±0.07) mm.

The final test of pollinator effectiveness, whether flowers visited by the different pollinators set fruit, provided clear results. Placing potted plants in the field, observing visits, bagging, tagging and following subsequent fruit set showed that only flowers visited by long-tongued bees set fruit following a single visit. Of the 44 flowers visited by long-tongued bees, 16 (36.4 %) set fruit. None of the flowers visited by the other pollinator groups (4 by non-skippers butterflies, 19 by skippers and 2 by short-tongued bees) produced any fruit. Fruit set differed significantly among pollinator groups (Kruskal–Wallis test, $\chi_{3}^{2}=111.7,$ *P* = 0.009, *n* = 69). Though the sample sizes were small for non-skipper butterflies and short-tongued bees due to their lower rate of visitation (Table 2), we also found that these groups of visitors do not carry significant amounts of pollen on their proboscides; consequently, we conclude that these two groups of visitors are much less effective pollinators of *A. berteroi* than are long-tongued bees.

## Discussion

Pollination efficiency is a function of multiple interacting characters and behaviours, including flower shape and size as well as animal behaviour and morphology (Ollerton *et al*. 2007). In the pine rocklands of South Florida, *A. berteroi* is effectively pollinated by two native solitary bees (*Megachile georgica* and *Melissodes communis*). Though many other species visit the flowers, the ones that are the most frequent (i.e. skippers like *Polites baracoa*) neither carry nor deposit pollen.

Many studies of other plant–pollinator systems provide evidence that the morphological match between the pollination apparatus and the length of the proboscis is associated with pollination effectiveness (Inouye 1980; Waser *et al*. 1996; Castellanos *et al*. 2004; Moré *et al*. 2012; Miller *et al*. 2014). Our experiment with fishing lines of various thicknesses, however, suggests that the width of the proboscis of the pollinators of *A. berteroi* is more important than the length in determining pollen transfer efficiency.

Flowers in the Apocynaceae tend to be pollinated by long-tongued pollinators (Endress 1994), with many reports of butterflies and hawkmoths pollinating species of this family (Haber 1984; Darrault and Schlindwein 2005; Sugiura and Yamazaki 2005; Moré *et al*. 2007). We found, surprisingly, that skippers and non-skipper butterflies did not carry much pollen on their proboscides, nor did they deposit pollen on stigmas of *A. berteroi*: it appears that they were acting as nectar thieves. Both skippers and non-skipper butterflies have been described as nectar thieves in other systems (Adrienne *et al*. 1985). Hoc and Garcia (1999) found Lepidoptera to be nectar robbers for *Phaseolus vulgaris*. Castro *et al*. (2013) reported that for flowers of *Polygala vayredae*, several species of Lepidoptera behave as nectar thieves.

The complex flower morphology of *A. berteroi* is similar to the morphology described for other Apocynaceae (Barrios and Koptur 2011). The flowers restrict access to only those visitors with mouthparts long enough to reach the nectar at the base of the floral tube. Furthermore, the sugar concentration of the nectar (30–67 %) is within the range of values reported for flowers pollinated by long-tongued bees (∼40 %; Proctor *et al*. 1996). Although Pascarella *et al*. (2001) stated that *A. berteroi* is visited exclusively by Lepidoptera, our field observations showed that long-tongued bees were common floral visitors, as well as skippers. Skippers were the most frequent and constant visitors, often visiting numerous flowers of the same species in a row.

We have observed that skippers and non-skipper butterflies often revisit the same flowers, while long-tongued bees rarely return to a previously visited flower. Insects revisiting the same flowers could have negative consequences, as *A. berteroi* has a late-acting self-incompatibility mechanism (Barrios and Koptur 2011). In many self-incompatible Apocynaceae, flower revisitation increases the probability that self-pollen is deposited onto the stigma, leading to ovule and fruit abortion (Lipow and Wyatt 1999, 2000; Wyatt *et al*. 2000; Wyatt and Lipow 2007). Abortion interferes with ovules in those fruits developing from cross-pollination and wastes those potential progeny (Lipow and Wyatt 1999; Lopes and Machado 1999).

The principal pollinators of *A. berteroi* in the pine rocklands of south Florida are two native bees; bee pollination (mostly by *Euglossine* bees) has been previously reported for this family (Lopes and Machado 1999; de Moura *et al*. 2011). Even though their proboscides are slightly shorter than the length of the narrow portion of the floral tube (i.e. 6 mm, Barrios and Koptur 2011), long-tongued bees carry large quantities of pollen on their proboscides. Evidently, long-tongued bees push their mouthparts firmly against the anthers in their effort to reach the nectar at the bottom of the floral tube and thereby pick up much more pollen on the wide proboscis base than the narrow, longer mouthparts of Lepidoptera that apparently miss the reproductive parts of the flowers entirely. Many studies have highlighted a close match between the length of the flowers and the length of pollinator mouthparts of members of the family Apocynaceae (Endress 1994; Proctor *et al*. 1996; Lopes and Machado 1999; Darrault and Schlindwein 2005; Moré *et al*. 2007). In contrast, we observed no association between the lengths of the proboscis of the pollinators with pollen removal, but we did find a correspondence between the width of pollinator mouthparts and pollination efficiency.

Our results contrast with the findings of Moré *et al*. (2007) and de Araújo *et al*. (2014), who reported that flowers of *Mandevilla* spp. were pollinated exclusively by pollinators with long, thin proboscides. Additionally, de Araújo *et al*. (2014) reported that *Agraulis vanillae* and *Ascia monuste* are the effective pollinators of *Mandevilla tenuifolia*; coincidently, these two taxa were the same non-skipper butterfly visitors of *A. berteroi* in southern Florida. The floral morphology of *A. berteroi*, however, with a relatively wide and large throat (bell) and a short narrow basal tube, allows visitors with mouthparts shorter than those of butterflies to enter the flowers, reach the nectar and contact the pollen-bearing, glue-applying and receptive portions of the stigma in the process. In our study, the long-tongued bees' heads were wider than the apical portion of the pollen chamber, causing the bees to touch the reproductive parts of the flowers; short-tongued bees, with their thinner heads, entered more deeply into the corolla, but did not contact the gynostegium. The thicker fishing line used to simulate the size of the mouthparts of the long-tongued bees removed twice as much pollen as the thinner lines, and we observed a similar pattern with our pollen deposition experiments, in which the thicker fishing line touched the stigmatic surface much more extensively than thinner ones. Using a similar approach, Darrault and Schlindwein (2005) observed that proboscis width played an important role in pollen transfer efficiency in *Hancornia speciosa* (Apocynaceae).

## Conclusions

By looking more closely into the mechanics of pollen removal and deposition, and by allowing single visits by the four groups of visitors, our results demonstrate that pollen removal ability, and single-visit fruit set data, can be more important for determining effective pollinators than simple visitation frequency. The relatively shorter mouthparts of long­tongued bees appear to be more than adequate to effectively garner nectar and effectively transport pollen, despite previous assumptions to the contrary.

## Sources of Funding

Funding was provided to B.B.R. by The Florida Native Plant Society (2008 Endowment Research Grant), FIU Kelly Scholarships (2008, 2009, 2012, 2013), the Catherine H. Beattie Fellowship (2009) from The Garden Club of America and a Florida International University Doctoral Evidence Acquisition Fellowship (2014).

## Contributions by the Authors

B.B.R. and S.K. were involved in the study conception and design. B.B.R. designed the experiments, analysed data and wrote the manuscript. B.B.R., S.R.P. and A.S. participate in the data collection. S.K. was involved in editing the manuscript. All authors had intellectual input into the project.

## Conflict of Interest Statement

None declared.
